# High carbon emissions from thermokarst lakes of Western Siberia

**DOI:** 10.1038/s41467-019-09592-1

**Published:** 2019-04-04

**Authors:** S. Serikova, O. S. Pokrovsky, H. Laudon, I. V. Krickov, A. G. Lim, R. M. Manasypov, J. Karlsson

**Affiliations:** 1 Climate Impacts Research Centre (CIRC), Department of Ecology and Environmental Science, Umeå University, Linnaeus väg 6, 901 87 Umeå, Sweden; 2 GET UMR 5563 CNRS, Geoscience and Environment, University of Toulouse, 14 Avenue Edouard Belin, 31400 Toulouse, France; 3 Department of Forest Ecology and Management, The Swedish University of Agricultural Sciences, Skogsmarksgränd, 901 83 Umeå, Sweden; 4 BIO-GEO-CLIM Laboratory, Tomsk State University, Lenina 36, 634050 Tomsk, Russia; 5 N. Laverov Federal Center for Integrated Arctic Research, IEPS, Russian Academy of Sciences, Nab. Severnoi Dviny 23, Arkhangelsk, 163000 Russia

## Abstract

The Western Siberia Lowland (WSL), the world’s largest permafrost peatland, is of importance for understanding the high-latitude carbon (C) cycle and its response to climate change. Warming temperatures increase permafrost thaw and production of greenhouse gases. Also, permafrost thaw leads to the formation of lakes which are hotspots for atmospheric C emissions. Although lakes occupy ~6% of WSL, lake C emissions from WSL remain poorly quantified. Here we show high C emissions from lakes across all permafrost zones of WSL. The C emissions were especially high in shoulder seasons and in colder permafrost-rich regions. The total C emission from permafrost-affected lakes of WSL equals ~12 ± 2.6 Tg C yr^−1^ and is 2-times greater than region’s C export to the Arctic coast. The results show that C emission from WSL lakes is a significant component in the high-latitude C cycle, but also suggest that C emission may decrease with warming.

## Abstract (web summary)

The Western Siberia Lowland (WSL) is the world’s largest frozen peatland complex, however carbon emissions (CO_2_+CH_4_) from lakes in this region remain unknown. Here, the authors sample 76 lakes and show high carbon emissions from lakes across all permafrost zones in the WSL.

## Introduction

At high-latitudes, temperatures have risen twice as fast as the global average^1^, resulting in widespread permafrost thaw and release of organic carbon (OC) to adjacent waters^2^. Permafrost thaw also leads to the formation of lakes and ponds of various size (hereafter referred to as lakes) that currently cover ~7% of the permafrost-affected areas^3^ of the Earth. Because of its relatively high lability^4^, OC released from thawing permafrost can be mineralized and emitted from the water surface of lakes in the form of carbon dioxide (CO_2_) and methane (CH_4_), two potent greenhouse gases. Outgassing of both CO_2_ and CH_4_ from lakes is of significance to the global carbon (C) cycle^5,6^. Yet quantifying C emissions from lakes remains challenging, especially in permafrost areas with numerous remote lakes of diverse size.

It has been suggested that climate-induced warming and concurrent permafrost thaw will increase C emissions^5,7^ from permafrost-affected lakes, mainly as a result of increased terrestrial OC supply and rising water temperatures. . Quantifying C emissions from permafrost-affected lakes is therefore important for providing accurate assessments of the role of climate and permafrost thaw on lake C evasion, especially at high latitudes, where the most dramatic changes due to warming are under way.

Although measurements of C emissions from high-latitude lakes have interested scientists for past decades^8–11^, direct measurements of C emissions from permafrost-affected lakes are rare^7^. Available data suggest that high-latitude lakes, including permafrost-affected lakes, represent a net source of C into the atmosphere^8–11^ and are recognized as important contributors to regional and global climate^12^. These estimates, however, often do not cover seasonal variability in lake C emissions, that if neglected could result in major errors in quantifying annual lake C contribution to atmospheric C budget^8^. Further, available data on C emissions from lakes are geographically biased as they have only covered small areas^8–11^, that do not allow assessments of the role of climate and permafrost on lake C emissions at a larger scale. Such lack of data for annual lake C emissions across a complete permafrost gradient implies that the role of lakes in permafrost-climate feedback is poorly constrained and can lead to large uncertainties when predicting climate change impacts following permafrost thaw.

The Western Siberia Lowland (WSL), the largest frozen peatland region of the world (~1.3 million km^2^) containing ~70 Pg C^13,14^, is of particular interest for understanding climate-induced changes in the C cycle at high latitudes. Recent studies stress that permafrost in WSL is vulnerable to thaw^15^ and has been actively degrading over past decades^15^. Lakes represent a common landscape feature in WSL and are formed mainly due to thawing of permafrost^16,17^ (i.e., thermokarst activity). These thermokarst lakes can range in size from small ponds to large lakes, but are typically shallow^16,18,19^ compared to Alaskan^9,11^ and Canadian^7,10^ thermokarst lakes of similar size. Yet, there are only few snapshot measurements of CO_2_ and CH_4_ concentrations^5,17,20–22^ and no estimates of atmospheric C exchange for lakes across WSL, implying that their role in C cycle is poorly constrained.

In this study, we quantified the atmospheric C emission (CO_2_ + diffusive CH_4_) from 76 thermokarst lakes located across a latitudinal gradient from 62 to 67°N of WSL. The North–South gradient encompasses major differences in mean annual air temperature (MAAT; from −1 to −5 °C) and permafrost extent (from isolated to continuous permafrost zone). The 76 lakes spanned the size range from 115 to 1,237,000 m^2^ and were sampled 3 times over the open water season of 2016; after ice-off in spring, in summer and before the development of ice cover in autumn. We measured concentrations and fluxes (using floating chambers) of CO_2_ and diffusive CH_4_, and calculated the annual C emission by multiplying mean daily C fluxes with the number of ice-free days for each lake.

We find that C emissions were especially high in shoulder seasons and in colder permafrost-rich regions, and estimate that the total C emission from WSL lakes is 2-times greater than region’s C export to the Arctic coast. Such finding suggests that WSL lakes play an important role in the high-latitude C cycle.

## Results

### Seasonal lake C fluxes

**Fig. 1:**
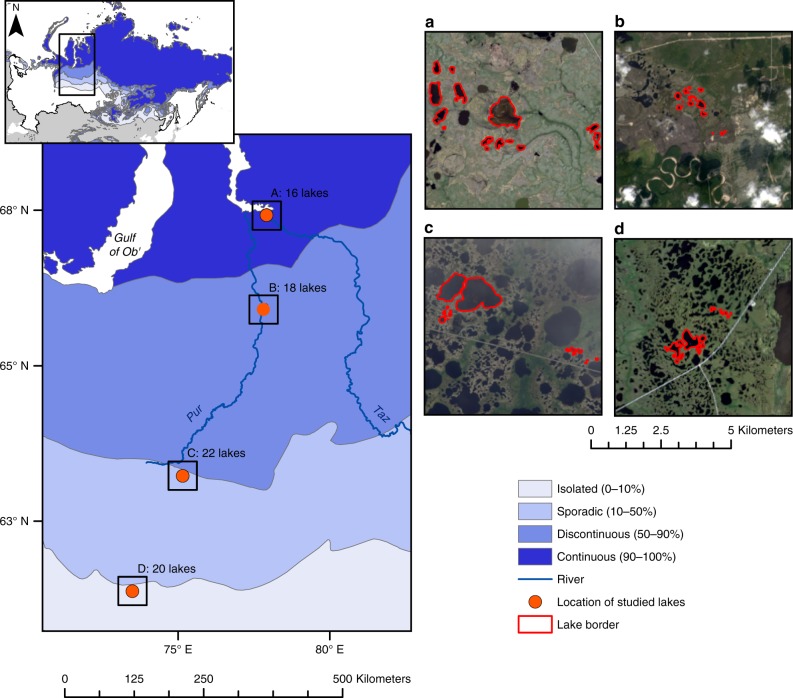
Map of the study area in the Western Siberia Lowland, Russia. Blue shading represents percent permafrost extent in the area of Western Siberia Lowland based on freely-available shapefiles from Brown et al.^58^. Orange dots indicate the location of the studied sites and red lines show shorelines of the studied lakes. Panel (**a**) refers to the site in the continuous permafrost zone, panel (**b**) to the discontinuous, panel (**c**) to the sporadic permafrost zone whereas panel (**d**) to the isolated permafrost zone. For details on satellite images acquisition see Ancillary data

**Fig. 2:**
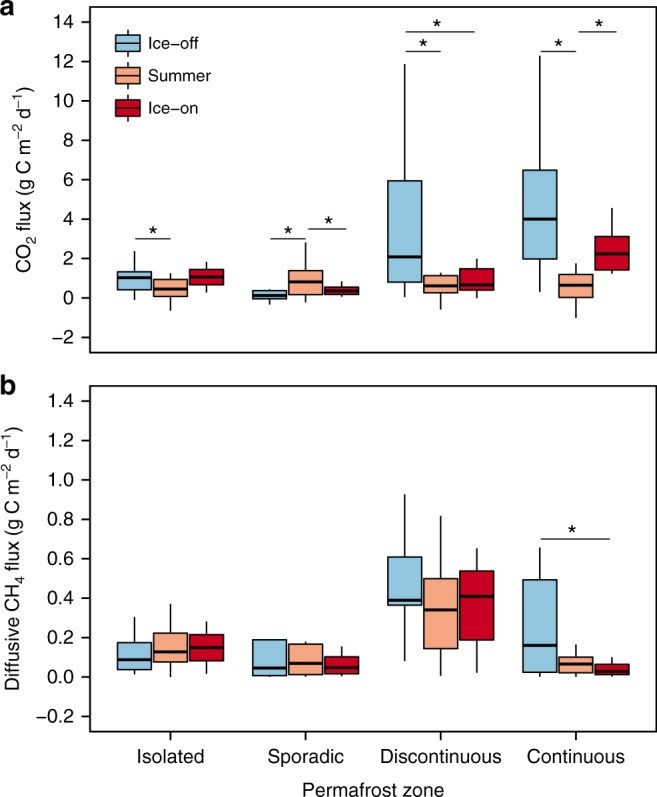
Seasonal CO_2_ flux and diffusive CH_4_ flux across different permafrost zones. The panel (**a**) refers to CO_2_ flux whereas panel (**b**) refers to diffusive CH_4_ flux. Boxes are bound by 25th and 75th percentiles, whiskers show 1.5 interquartile range. Solid line represents median values while the star signifies statistically significant differences. Positive values indicate outward flux from the lakes into the atmosphere. We removed 12 outliers on panel (**a**) and 14 outliers on panel (**b**) to visually improve the graph, but used the complete dataset for statistical analyses. For sample size see Supplementary Table 1

85% of all studied lakes across different permafrost zones of WSL (Fig. 1) were supersaturated in *p*CO_2_ (1044 ± 554 ppmv, mean ± interquartile range, IQR) and all lakes were supersaturated in *p*CH_4_ (20.4 ± 21.8 ppmv) (Supplementary Table 1). The CO_2_ fluxes varied among the permafrost zones (1.7 ± 1.7 g C m^−2^ d^−1^) and showed strong seasonal differences in all zones, whereas diffusive CH_4_ fluxes (0.2 ± 0.2 g C m^−2^ d^−1^) did vary among the seasons only in the continuous permafrost zone (Fig. 2, Supplementary Tables 1–3). Overall, the C fluxes were dominated by CO_2_, which constituted on average 88 (±12)% of total C flux across seasons. However, we only estimated diffusive CH_4_ fluxes, and not ebullition that can exceed diffusive CH_4_ fluxes by up to 3-fold in thermokarst lakes^5,22^ and therefore can raise the overall contribution of CH_4_ fluxes to the total C flux. Also, considering the ~30-fold stronger global warming potential of CH_4_ vs. CO_2_ (GWP_100_ = 28)^2,23^ the C flux expressed in CO_2_ equivalents from WSL lakes can increase on average 15-times, further emphasizing the importance of lake feedback effects on the climate system.

Comparing our values to daily rates reported from permafrost-affected lakes in other high-latitude areas^5,7–9^ suggests that our estimates for both mean daily rates of CO_2_ and diffusive CH_4_ fluxes are 1.5–14-times greater. Importantly, the C fluxes from WSL lakes in spring and autumn were on average 2-times greater than during summer in all permafrost zones apart from isolated permafrost zone. Such strong seasonality has been shown for other lakes and is largely explained by spring release of gas accumulated under ice in winter and autumn release of gas accumulated in hypolimnion over summer^24–27^. However, bottom freezing in winter^19^ and lack of stable thermal stratification^20^ in summer of WSL lakes implies that these storage fluxes are likely not the main factors for the observed strong seasonality. Irrespectively of the underlying mechanisms, the seasonality in C fluxes from WSL lakes emphasizes the need for integrating spring and autumn C fluxes estimates for accurately assessing annual lake C emission.

### Annual lake C emission

**Fig. 3:**
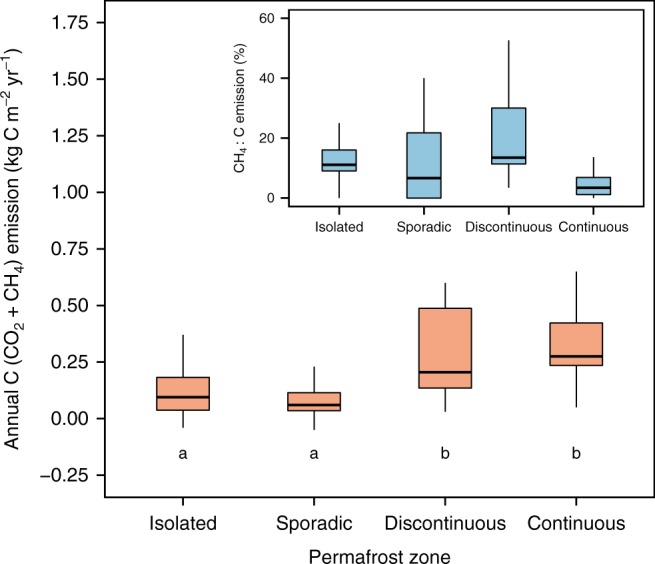
Annual C (CO_2_ + diffusive CH_4_) emission across different permafrost zones. Annual C emission is greater in colder permafrost-rich regions compared to warmer permafrost-poor regions (see Fig. 1 for the geographical location of different permafrost zones). The inset shows the percent of diffusive CH_4_ emission in annual C emission across different permafrost zones. Boxes are bound by 25th and 75th percentiles, whiskers show 1.5 interquartile range. Solid line represents median values. Positive values indicate outward flux from the lakes into the atmosphere. Permafrost zones that share a letter are not significantly different. We removed 3 outliers on the main plot and 5 outliers on the inset to visually improve the graph, but used the complete dataset for statistical analyses. For sample size see Supplementary Table 4

The total annual diffusive C emission (CO_2_ + diffusive CH_4_) from WSL lakes shows strong differences across different permafrost zones (range: 0.1 (±0.1) to 0.3 (±0.1) kg C m^−2^ yr^−1^, *H* = 22.59, *P* < 0.05, Fig. 3), with 2–3 times higher lake C emission in the most northern zones with discontinuous and continuous permafrost compared to the southern zones with isolated and sporadic permafrost (Supplementary Table 4). Although there is a limited number of studies to compare with, the annual CO_2_ and diffusive CH_4_ emission from WSL lakes are 1.5–5-times greater than annual CO_2_^7^ and diffusive CH_4_ emission^5^ from other permafrost-affected lakes across the Arctic, but are similar to values reported for other thermokarst lakes in Eastern Siberia^28^ and Alaska^11^. Also, the annual diffusive CH_4_ emission from WSL lakes is similar to annual whole-lake CH_4_ ebullition from yedoma lakes^29^ in Eastern Siberia.

## Discussion

The fact that C emission from WSL lakes is higher in colder permafrost-rich regions is at odds with findings from studies of boreal and arctic lakes and also the general understanding of the impact of warming on lake C cycling, where warming-induced OC loading and enhanced metabolism lead to increased CO_2_ and especially CH_4_ production^2,30^. While recent assessment of C emission from high-latitude lakes^31^ has shown a decline in C evasion from lakes with lower MAAT, the WSL lakes show an opposite pattern with higher C emission from permafrost-rich areas, where lake water temperatures are generally colder (*H* = 51.766, *P* < 0.05). Further, there was no trend of increasing fraction of CH_4_:CO_2_ emission with warmer water temperatures (log_10_-transformed, *n* = 62, *R*^2^ = 0.00, *F*_1,60_ = 0.146, *P* > 0.05). And despite the relatively high concentrations of dissolved OC in lake water (15.7 ± 7.7 mg L^−1^), presumably of mainly terrestrial origin^19^, there was no relationship with total lake C emission (*n* = 73, *R*^2^ = 0.00, *F*_1,71_ = 1.438, *P* > 0.05, Supplementary Table 5). Neither did we find any dependence of total lake C emission across permafrost zones of WSL to other factors (i.e., lake area, lake depth, nutrient concentrations, SUVA_254_, O_2_ concentration, Supplementary Table 5) that could potentially affect CO_2_ production and emission from lakes. Taken together, the temporal and spatial patterns in C emission of WSL lakes suggest that lake C emission is controlled differently compared to other regions, unraveling a complex interaction between climate and permafrost and their combined regulation of C emission from WSL lakes.

One potential explanation for the observed patterns is the delivery of CO_2_ from the surrounding soils, which is higher in permafrost-rich vs. permafrost-poor regions of WSL^32^, and is expected to result in higher C emission from small vs. large lakes^33^. However, the lack of dependence of C emission on lake size suggests that variation in soil CO_2_ production and export alone cannot explain the observed patterns and that other factors are likely more important. A specific feature of WSL lakes compared to many other lakes in permafrost-affected regions are their shallow depths, rarely deeper than 1–1.5 m (0.8 ± 0.7 m), found even in lakes large in size (log_10_-transformed, *n* = 74, *R*^2^ = 0.22, *F*_1,72_ = 21.16, *P* < 0.05). It is plausible that owing to the overall flat terrain of WSL (with widespread peat mounds and hollows), these lakes also have relatively small catchments^18^. Together, this implies that sediments play a larger role in lake C cycling compared to deeper lakes with larger catchments where lateral inputs of C and its processing in the water column dominate. Shallow water depth also enhances wind-induced mixing, and thus keeps water and sediment surface warm and oxic (Supplementary Table 6), further facilitating aerobic respiration in sediments. Accordingly, published rates of net ecosystem production of CO_2_ in the water column of lakes and ponds within the discontinuous permafrost zone of WSL^21,34^ are low (~0.3–0.5 g C m^−2^ d^−1^) compared to C fluxes (1.7 ± 1.7 g C m^−2^ d^−1^) measured in this study. The sediments are mainly composed of organic detritus from flooded peat bogs and it has been shown that a major part of this peat is mineralized in sediments of WSL lakes^35^ over the course of lake development. Thus, if sediments play an important role in lake C cycling it is also likely that observed latitudinal patterns in C emission are largely governed by a higher availability of OC for mineralization of recently thawed lake sediments in the north compared to in the south, similar to what has been reported for availability of thawed soil OC for mineralization in lake^2^ and river water^4,36,37^. Also, given the shallow nature of these lakes, photosynthetic CO_2_ fixation is likely predominately benthic and mainly constrained by light conditions^38^ thus decreasing with the shorter ice-free season in the north compared to the south. Another potential mechanism is variability in photomineralization, but recent studies suggest photomineralization to be negligible for the net OC mineralization in permafrost-affected lakes located in this area^39^. Nevertheless, the relative importance of greenhouse gas sources and sinks in WSL lakes remains a knowledge gap and more in-depth studies are needed to infer a better mechanistic understanding of controls of WSL lake C evasion.

Up-scaling our results to the permafrost-affected area of WSL (60–74°N, ~1.3 million km^2^) shows that WSL lakes at present emit ~12 ± 2.6 Tg C yr^−1^. The C emission from WSL lakes alone is half of the C emission from lakes reported earlier for the Artic region between 63 and 90°N^31^, and 2-times greater than annual river OC export^40–42^ from WSL to the Arctic Ocean. Although these estimates contain uncertainties, the C emission is 3-times greater than total CH_4_ emission from yedoma lakes in Eastern Siberia^29^ suggesting that thermokarst lakes of WSL are important contributors to net C evasion from high latitude lakes and that previous assessment of northern lake C emission^31^ is likely underestimated. In our up-scaling we assumed a lake coverage of 6%^16,17^, yet recent estimates of certain areas within WSL suggest that wet area extent (flood zones + lakes) can have a strong temporal variation and reach up to ~70%^43^ during spring and autumn. This implies that WSL lake C emission is likely higher than our conservative estimate, further emphasizing the need to capture variability in both flux rates and thermokarst lake area across the Arctic.

Our results stress the need to integrate lake C emission estimates to accurately predict permafrost feedback to a warming climate. Given the vast terrestrial stock of WSL (~70 Pg C)^13,14^, even a minor change in lateral OC export is likely to considerably alter C emission from WSL lakes. Further warming will result in a northward shift of permafrost zones^44^ and their subsequent replacement with permafrost-free regions. Interestingly, by substituting space-for-time our results suggest that this would lead to a decrease in C emission from WSL lakes. However, such space-for-time substitution approach is likely unable to fully capture impacts of new environmental conditions following warming and permafrost thaw on lake C cycling. We therefore conclude that understanding this complex interaction between climate and permafrost, especially in such areas as WSL, is fundamental when predicting future C cycle and call for more work and alternative approaches to test such predictive models.

## Methods

### Sampling sites

The studied region is the Western Siberia Lowland (WSL, Russia). WSL has a humid semi-continental climate^17^ with MAAT ranging from ca. −0.5 °C in the south to −9.5 °C in the north, and mean annual precipitation ranging from 600 to 350 mm yr^−1^, accordingly^42,45^. The region is characterized by a low and flat relief (0–200 masl)^46^ and is dominated by Pliocene sands and clays^42^. The combination of both cold temperatures and flat relief have enabled accumulation of ~70 Pg of carbon in region’s extensive peatlands over past 11,000 years^13,14^. More than half of the region is influenced by permafrost, with 15% of WSL covered with continuous and 39% covered with discontinuous, sporadic, and isolated permafrost^13,47^. We sampled 76 lakes of different size across permafrost gradient of WSL (between 62°N and 67°N) (Fig. 1), with 20 lakes located in the isolated zone, 22 in sporadic, 18 in discontinuous, and 16 in continuous, respectively. The size of the sampled lakes ranged from 115 to 1,237,000 m^2^. We visited all sites in 2016 at ice-off event (20th of May–13th of June), in the middle of the summer (9th–24th of August) and just before lakes established ice cover (26th of September–8th of October). We designed our three sampling campaigns in a way that they followed the natural propagation of seasons across WSL, by starting the spring sampling campaign from the south, whereas summer and autumn campaigns from the north.

### Sampling procedure

Sampling of surface water was carried out from a boat at the deepest part of lakes in lakes smaller than 10,000 m^2^, while samples from lakes larger than 10,000 m^2^ were taken ~200–300 m offshore. At each location we measured water temperature and dissolved oxygen saturation (YSI ProODO Handheld Optical Dissolved Oxygen meter), pH and specific conductivity (WTW Multi 3320 multiparameter) as well as water depth (Cole-Parmer), air temperature, and atmospheric pressure (Silva). Dissolved oxygen saturation was measured by submerging the probe at 50 cm interval until reaching lake sediment interface. Water samples for DOC, DIC, nutrients, and dissolved CH_4_ were collected and analyzed following methods described elsewhere^18,19,48^. Sampling for partial pressure of CO_2_ (*p*CO_2_) in situ was conducted following the procedure described in our previous work^48^. We also measured ultraviolet absorbance at 245 nm (UV_245_) (Bruker CARY-50 UV-VIS) and calculated specific ultraviolet absorbance (SUVA_254_) of the sampled water following conversion of UV_245_ to UV_254_^49^.

### C fluxes calculations

CO_2_ fluxes were measured with small lightweight^50^ (~30–32 cm in diameter, ~270–300 g) floating chambers^50,51^ equipped with non-dispersive infrared CO_2_ logger (ELG, SenseAir) and calculated following methods described in literature^48,51,52^. We calibrated CO_2_ loggers in the lab against pure N_2_ before each sampling campaign. We placed 2–6 chambers per lake along the transects from the shore to the center of the lake, if the lake size allowed. If the lake was sufficiently big to prevent us from reaching its center, we distributed the chambers along the transects from the shore to approximately 100–200 m offshore. The CO_2_ accumulation rate inside each chamber was recorded continuously at 300 s interval. We used first 1–2.5 h of measurements for computing CO_2_ accumulation rate inside each chamber by linear regression. Although 87% of 487 measurements had a linear increase with *R*^2^ ≥ 0.76, 13% of the measurements had a linear increase with *R*^2^ ≤ 0.75. These measurements were retained only if the average *R*^2^ between the replicates was greater or equal to 0.55, discarding those measurements that did not meet this requirement. Also, while 71% of all chamber readings across all seasons had linear increase, 29% of measurements recorded a persistent decrease in CO_2_ accumulation rate. We interpreted these measurements as CO_2_ uptake and retained the values if *p*CO_2_ concentration in the water of the respective lake was close to or below atmospheric equilibrium set to 404.2 ppm^48,51,52^, mean annual *p*CO_2_ concentration in the air for 2016 (Mauna Loa Observatory fttp://aftp.cmdl.noaa.gov/products/trends/co2/co2_annmean_mlo.txt). We calculated instantaneous diffusive CH_4_ fluxes using concentrations of dissolved CH_4_ in the water and air–water equilibrium *p*CH_4_ concentration of 1.8 ppm, mean annual *p*CH_4_ concentration in the air for 2016 (Mauna Loa Observatory fttp://aftp.cmdl.noaa.gov/products/trends/ch4/ch4_annmean_gl.txt). We further averaged both CO_2_ and diffusive CH_4_ fluxes from all chambers for each of the lakes and summed them up to estimate total C fluxes from the respective lakes.

### Chamber C fluxes vs. wind-based model C fluxes

Although chamber measurements can potentially increase C fluxes due to turbulence at chambers’ edge^53^, they are still the most accurate tool for obtaining direct flux measurements^50^ in such remote locations as Western Siberia. To compare our results obtained with chambers with wind-based models we computed C fluxes using published relationship from Cole and Caraco^54^ (Eq. (1)) and Vachon and Prairie^55^ (Eq. (2)):

1 $$k_{600} = 2.07 + 0.215 \cdot U_{10}^{1.7}$$

2 $$k_{600} = 2.51\left( \!\!\pm 0.99 \right) + 1.48\left(\!\!\pm 0.34 \right) \cdot U_{10} + 0.39\left( \!\!\pm 0.08 \right) \cdot U_{10} \cdot \log_{10}\! {\mathrm{LA}}$$

where $U_{10}$ is the wind speed at 10 m height (monthly average) and LA is lake area in km^2^. We used mean wind speed recorded at the nearest meteostation during the time the chambers were deployed at each of our sites, while we used the lake area representing the conditions of summer sampling (for details see Ancillary data). The results suggest a relationship between measured and modeled fluxes, but also show that fluxes calculated with wind-based models are on average 3 and 4-times lower than measured CO_2_ and CH_4_ fluxes, respectively (Supplementary Figure 1). This discrepancy between modeled and measured values may be explained by the remoteness of the studied lakes in relation to the respective meteostations (~20–70 km from the lakes), and points out the need for future studies relying on wind-based models for WSL lakes to validate these findings with more precise local wind data.

### Ancillary data

We quantified average wind speed, mean annual temperature, and precipitation for 2016 at each of the studied sites based on meteorological records available at https://rp5.ru/. Since we followed our sampled lakes from ice-off event until the establishment of ice cover, we used the number of ice-off days from our field observations. We quantified water surface area of the respective lakes by analyzing satellite images of the studied sites. We used Landsat 8 scenes freely-available at https://remotepixel.ca/projects/satellitesearch.html. Due to the substantial cloud cover (>50%) of the images matching our ice-on and ice-off sampling campaigns, we used four images for 19th of June and 14th of July (LC81570132016171LGN00_B432, LC81560142016196LGN00_B432, LC81560152016196LGN00_B432, LC81570162016171LGN00_B432) that had cloud cover within 10–30% and reflected the conditions of summer sampling. We calculated water surface areas of each of the studied lakes by manually drawing polygons around each lake within the studied sites using ArcMap 10.5. If the lakes were not visible on the Landsat 8 scene, we first drew the polygon of the respective lake in Google Earth, quantified its area and then drew a polygon of similar size in the Landsat 8 scene at the location matching lake’s GPS coordinates. We further checked our lake water surface areas against water surface areas of GLOWABO global lake database^56^, where information for 16 out of 76 lakes sampled in this study is available. Our quantified lake water surface areas were in good agreement with those reported in GLOWABO (log_10_-transformed, *n* = 16, *R*^2^ = 0.94, *F*_1,14_ = 247.9, *P* < 0.05)

### Upscaling

We estimated present lake C emission from the permafrost-affected area of WSL^16,17^ (60–74°N) by multiplying total lake area of 6%^16,17^ with mean lake C emission measured in our study. We assumed 15% uncertainty in estimates of both total lake area and lake C emission and propagated our error following equation:

3 $$\delta R = \left| R \right|\sqrt {\left( {\frac{{\delta x}}{x}} \right)^2 + \left( {\frac{{\delta y}}{y}} \right)^2}$$

where $\delta R$ is the uncertainty, $R$ is a result of multiplication of total lake area with mean lake C emission, while $\delta x$ and $\delta y$ are 15% uncertainty estimates for total lake area, $x$, and mean lake C emission, $y$, respectively. We further estimated present C emission from WSL lakes based on lake coverage available in literature^56,57^ and compiled different estimates in Supplementary Table 7.

### Statistical analysis

All statistical analyses and calculations were performed in RStudio statistical software (Version 1.0.44, RStudio, Inc., 〈www.r-project.org〉). Prior to statistical analyses, we grouped sampled lakes based on the sampling location in four groups representing different permafrost zones: isolated (62°N, *n* = 20), sporadic (63°N, *n* = 22), discontinuous (66°N, *n* = 18), and continuous (67°N, *n* = 16). We further assessed homogeneity of variances between the groups by using either parametric Bartlett and non-parametric Fligner–Killeen tests, or by randomly subsampling 10 lakes within each permafrost zone to balance our study design. If the variances between the groups were homogenous and the data was normally distributed, we used one-way analysis of variance (ANOVA) with Tukey’s HSD post-hoc comparisons to investigate differences in annual C emission and mean summer concentrations of variables among different permafrost zones. If the variances between the groups were not homogeneous, we used a non-parametric alternative of Kruskal–Wallis test together with Pairwise Wilcox test (Holm adjustment).

When analyzing the seasonal differences in variables among different permafrost zones, we classified sampled lakes in four different classes based on water surface area: thaw ponds (<499 m^2^, *n* = 24), thermokarst lakes (500–9999 m^2^, *n* = 90), mature lakes (10,000–999,999 m^2^, *n* = 90) and massive lakes (>999,999 m^2^, *n* = 3). We further classified sampled lakes in five categories based on average water depth in different seasons: very shallow (<0.4 m, *n* = 38), shallow (0.5–0.9 m, *n* = 69), average (1–1.4 m, *n* = 46), deep (1.5–1.9 m, *n* = 14), and very deep (>1.9 m, *n* = 15). We used linear mixed effects models (*lme4* package) when analyzing two-way interactions of seasons and permafrost zones on the transformed per unit area daily CO_2_ fluxes, diffusive CH_4_ fluxes, surface water *p*CO_2_ and dissolved CH_4_ concentrations (Supplementary Tables 2–3, 8–10) along with other water chemistry variables. We used permafrost zones and seasons as fixed factors that are expected to have a systematic influence on the data while we allowed our sampled lakes to randomly vary inside permafrost zone groups, lake size classes and water depth categories as well as seasons inside permafrost zone groups to correct for possible effect of lake size and depth on the respective variable concentrations. In that way, we assumed that whatever the effects of permafrost extent and seasons are, they are going to be the same for all lakes sampled within each permafrost zone group. The best model fit was selected based on Akaike Information Criterion (AIC). We also performed contrasts analyses on the respective mixed effects models by constructing orthogonal contrasts to compare seasons between each other and avoid multiple comparisons (package *lsmeans*).

We further used simple linear regression when analyzing the relationship between variables of interest. Note that we report untransformed data in the text, figures, and tables. Because of non-normal distribution of the data, we use mean ± IQR when reporting uncertainty. All statistical tests used a significance level of 5% (*α* = 0.05) and were run on the complete dataset.

## Supplementary information

Supplementary Information

Description of Additional Supplementary Files

Supplementary Data 1

Supplementary Data 2

## Data Availability

All data generated and analyzed during this study are included in this published article (and its Supplementary Data 1–2).

## Code availability

Computer code for data analyses is available upon request.
