# Supplementary material for: High carbon emissions from thermokarst lakes of Western Siberia
Source: Nat Commun. 2019 Apr 4;10:1552. doi: 10.1038/s41467-019-09592-1 (PMC6449335; doi:10.1038/s41467-019-09592-1)
Supplement: Supplementary file 1 — Supplementary Information [file 41467_2019_9592_MOESM1_ESM.docx]

**Supplementary Information**

High carbon emissions from thermokarst lakes of Western Siberia

S. Serikova* et al.

*Correspondence to S.S. ([svetaserikova22@gmail.com](mailto:svetaserikova22@gmail.com))





**Supplementary Figure 1.** Measured CO_2_ flux and diffusive CH_4_ flux versus modelled. The (a, b) plots used wind-based model from Cole & Caraco^1^, whereas the (c, d) plots used wind-based model from Vachon & Prairie^2^. The red line indicates 1:1 line.

**Supplementary Table 1.** Mean seasonal surface water chemistry parameters and flux rates per unit of aquatic area (± interquartile range, IQR) across permafrost zones.

|  | **Permafrost zone** | | | | | | | | | | | |
| --- | --- | --- | --- | --- | --- | --- | --- | --- | --- | --- | --- | --- |
|  | **Isolated** | | | **Sporadic** | | | **Discontinuous** | | | **Continuous** | | |
|  | **Ice-off** | **Summer** | **Ice-on** | **Ice-off** | **Summer** | **Ice-on** | **Ice-off** | **Summer** | **Ice-on** | **Ice-off** | **Summer** | **Ice-on** |
| ***n*** | 20 | 18 | 18 | 22 | 16 | 17 | 18 | 16 | 16 | 16 | 16 | 16 |
| *p*CO_2_, ppmv | 708 (341) | 664 (335) | 583 (146) | 500 (103) | 1034 (465) | 1217 (167) | 1638  (1738) | 1317  (282) | 1476  (661) | 2023  (1133) | 730  (527) | 946  (396) |
| *p*CO_2_, μmol L^-1^ | 59.01 (28.3) | 55.36 (27.9) | 48.54 (12.1) | 41.64 (8.6) | 86.16 (38.7) | 101.34 (13.9) | 136.37 (144.7) | 109.71 (23.5) | 122.92 (55.0) | 168.44 (94.3) | 60.81 (43.8) | 78.80 (33.0) |
| ***n*** | 19 | 18 | 17 | 22 | 16 | 15 | 8 | 14 | 10 | 10 | 16 | 16 |
| *p*CH_4_, ppmv | 22.13 (18.3) | 21.72 (18.6) | 20.46 (18.1) | 10.61 (5.5) | 8.36  (5.2) | 11.53  (11.3) | 47.02  (35.3) | 32.25  (50.7) | 50.95  (45.6) | 19.51  (14.3) | 18.87  (13.6) | 10.24  (8.1) |
| *p*CH_4_, μmol L^-1^ | 1.37 (1.1) | 1.35 (1.1) | 1.27 (1.1) | 0.66 (0.3) | 0.52 (0.3) | 0.71 (0.7) | 2.93 (2.2) | 2.01 (3.1) | 3.17 (2.8) | 1.21 (0.8) | 1.17 (0.8) | 0.63 (0.5) |
| ***n*** | 19 | 18 | 2 | 19 | 16 | 10 | 18 | 16 | 16 | 16 | 16 | 16 |
| CO_2_ flux, g C m^-2^ d^-1^ | 1.24 (0.9) | 0.68 (0.8) | 1.05 (0.7) | 0.55  (0.4) | 0.93  (1.2) | 0.39  (0.3) | 4.45  (6.2) | 1.40  (0.8) | 1.47  (1.0) | 5.04  (4.5) | 0.52  (1.1) | 2.44  (1.7) |
| ***n*** | 16 | 14 | 2 | 16 | 13 | 7 | 7 | 10 | 12 | 15 | 15 | 16 |
| Diffusive CH_4_ flux, g C m^-2^ d^-1^ | 0.16 (0.1) | 0.18 (0.1) | 0.14 (0.1) | 0.15  (0.1) | 0.15  (0.1) | 0.11  (0.0) | 0.47  (0.2) | 0.37  (0.3) | 0.41  (0.3) | 0.30  (0.4) | 0.07  (0.0) | 0.12  (0.0) |
| ***n*** | 20 | 18 | 18 | 22 | 17 | 17 | 18 | 16 | 16 | 16 | 16 | 16 |
| DOC, mg L^-1^ | 9.64 (4.0) | 10.77 (4.5) | 11.34 (4.9) | 12.11  (7.0) | 27.37  (12.8) | 25.94  (13.5) | 10.90  (3.4) | 24.88  (9.9) | 24.09  (8.2) | 8.45  (4.2) | 13.49  (2.9) | 15.89  (8.3) |
| DOC, μmol L^-1^ | 9643 (4068) | 10779 (4523) | 11343 (4959) | 12116 (7081) | 27375 (12830) | 25942 (13530) | 10907 (3447) | 24883 (9987) | 24095 (8257) | 8456 (4296) | 13496 (2990) | 15891 (8330) |
| DIC, mg L^-1^ | 0.30 (0.1) | 0.27 (0.1) | 0.39 (0.1) | 0.31  (0.0) | 0.39  (0.1) | 0.49  (0.2) | 0.40  (0.0) | 0.42  (0.1) | 0.54  (0.3) | 1.40  (1.2) | 1.67  (1.7) | 1.81  (1.6) |
| DIC, μmol L^-1^ | 307.76 (109.0) | 273.61 (110.7) | 394.79 (191.4) | 317.16 (53.4) | 394.82 (166.9) | 495.70 (245.7) | 405.05 (44.4) | 423.59 (130.1) | 549.92 (320.6) | 1409.03 (1244.9) | 1670.66 (1789.6) | 1818.27 (1639.4) |
| ***n*** | 20 | 18 | 18 | 22 | 16 | 17 | 18 | 16 | 16 | 16 | 16 | 16 |
| O_2_, μmol L^-1^ | 602 (36.2) | 563 (18.5) | 801 (27.5) | 719  (87.9) | 597  (40.3) | 854  (55.0) | 627  (101.8) | 576  (20.9) | 752  (102.0) | 628  (85.0) | 605  (31.0) | 693  (29.8) |
| O_2_, % | 106 (7.0) | 101 (3.1) | 98 (3.0) | 98  (3.6) | 100  (6.4) | 101  (8.0) | 92  (15.2) | 100  (5.1) | 93  (13.5) | 93  (25.3) | 101  (7.2) | 94  (4.2) |
| ***n*** | 19 | 18 | 15 | 17 | 17 | 12 | 18 | 15 | 15 | 14 | 15 | 15 |
| PO_4_, μg P L^-1^ | 1.18 (1.9) | 1.65 (1.0) | 1.66 (1.2) | 2.37  (1.6) | 2.36  (2.3) | 3.30  (3.7) | 1.81  (0.7) | 5.51  (3.0) | 1.79  (2.3) | 1.94  (1.7) | 4.11  (2.4) | 2.42  (1.3) |
| NO_3_, μg N L^-1^ | 10.82 (9.2) | 3.51 (1.5) | 16.24 (7.4) | 13.09  (16.7) | 4.02  (4.6) | 27.87  (19.9) | 2.27  (2.8) | 2.07  (1.7) | 2.32  (2.8) | 2.34  (3.9) | 0.69  (1.0) | 16.93  (20.3) |
| NH_4_, μg N L^-1^ | 102.6 (135.3) | 17.1 (6.0) | 38.9 (10.4) | 97.5  (155.8) | 105.1  (118.8) | 347.7  (534.2) | 79.4  (81.2) | 54.1  (59.1) | 164.1  (83.9) | 9.2  (7.1) | 8.7  (4.6) | 23.0  (14.6) |
| DIN, μg L^-1^ | 113.5 (155.8) | 20.6 (7.5) | 55.1  (16.2) | 110.6  (160.4) | 109.2  (133.0) | 375.6  (627.0) | 81.7  (81.1) | 56.2  (62.0) | 166.4  (85.1) | 11.6  (10.0) | 9.4  (5.8) | 39.9  (41.3) |
| ***n*** | 19 | 18 | 18 | 22 | 16 | 17 | 17 | 16 | 16 | 16 | 16 | 16 |
| TP, μg L^-1^ | 10.49 (6.2) | 11.67 (9.3) | 8.53 (5.4) | 18.87  (10.5) | 33.75  (12.9) | 29.09  (19.3) | 28.53  (10.8) | 72.85  (34.2) | 53.28  (27.5) | 32.97  (9.3) | 49.11  (42.2) | 35.29  (22.5) |
| ***n*** | 20 | 18 | 18 | 22 | 17 | 17 | 18 | 16 | 16 | 16 | 16 | 16 |
| SUVA_254_ | 3.25 (0.5) | 2.89 (0.4) | 2.73 (0.3) | 3.16  (0.5) | 3.99  (0.6) | 3.87  (0.8) | 3.29  (0.8) | 3.84  (0.7) | 3.63  (0.6) | 3.74  (0.4) | 3.49  (0.5) | 3.32  (0.8) |
| ***n*** | 20 | 18 | 18 | 22 | 16 | 17 | 18 | 16 | 16 | 16 | 16 | 16 |
| pH | 4.55 (0.2) | 4.50 (0.1) | 4.50 (0.2) | 4.74  (0.5) | 4.13  (0.2) | 4.39  (0.3) | 4.58  (0.3) | 4.18  (0.2) | 4.58  (0.4) | 6.14  (0.6) | 6.99  (0.9) | 6.57  (0.7) |
| ***n*** | 20 | 18 | 18 | 22 | 16 | 17 | 18 | 16 | 16 | 16 | 16 | 15 |
| Conductivity, μS cm^-1^ | 13.70 (4.5) | 15.00 (4) | 18.03 (4.3) | 15.04  (9.0) | 24.00  (12.0) | 25.82  (7.8) | 13.27  (7.0) | 21.62  (11.0) | 23.05  (10.3) | 15.62  (8.5) | 21.18  (11.9) | 23.55  (10.8) |

**Supplementary Table 2.** Statistical results of orthogonal contrasts. Between-seasons comparison of CO_2_ fluxes (log_10_-transformed, n=155). The star indicates statistically significant difference at 0.05 level. For details on statistics see Statistical analysis.

| **Contrast** | **Permafrost zone** | **Estimate** | **SE** | **df** | **t.ratio** | **p.value** |
| --- | --- | --- | --- | --- | --- | --- |
| Ice-off_vs_Summer | Isolated | -0.342 | 0.146 | 103.830 | -2.345 | 0.021* |
| Ice-off_vs_Ice-on | Isolated | -0.431 | 0.331 | 126.214 | -1.304 | 0.195 |
| Summer_vs_Ice-on | Isolated | -0.089 | 0.331 | 125.538 | -0.269 | 0.788 |
| Ice-off_vs_Summer | Sporadic | 0.470 | 0.168 | 128.866 | 2.795 | 0.006* |
| Ice-off_vs_Ice-on | Sporadic | -0.119 | 0.216 | 130.973 | -0.551 | 0.583 |
| Summer_vs_Ice-on | Sporadic | -0.588 | 0.212 | 122.545 | -2.780 | 0.006* |
| Ice-off_vs_Summer | Discontinuous | -0.452 | 0.149 | 104.445 | -3.042 | 0.003* |
| Ice-off_vs_Ice-on | Discontinuous | -0.460 | 0.141 | 101.665 | -3.251 | 0.002* |
| Summer_vs_Ice-on | Discontinuous | -0.008 | 0.154 | 102.114 | -0.049 | 0.961 |
| Ice-off_vs_Summer | Continuous | -0.698 | 0.155 | 101.754 | -4.503 | 0.000* |
| Ice-off_vs_Ice-on | Continuous | -0.199 | 0.141 | 95.789 | -1.413 | 0.161 |
| Summer_vs_Ice-on | Continuous | 0.500 | 0.155 | 101.754 | 3.221 | 0.002* |

**Supplementary Table 3.** Statistical results of orthogonal contrasts. Between-seasons comparison of diffusive CH_4_ fluxes (log_10_-transformed, n=142). The star indicates statistically significant difference at 0.05 level. For details on statistics see Statistical analysis.

| **Contrast** | **Permafrost zone** | **Estimate** | **SE** | **df** | **t.ratio** | **p.value** |
| --- | --- | --- | --- | --- | --- | --- |
| Ice-off_vs_Summer | Isolated | 0.098 | 0.215 | 96.050 | 0.456 | 0.650 |
| Ice-off_vs_Ice-on | Isolated | -0.342 | 0.475 | 114.455 | -0.720 | 0.473 |
| Summer_vs_Ice-on | Isolated | -0.440 | 0.477 | 113.095 | -0.922 | 0.358 |
| Ice-off_vs_Summer | Sporadic | 0.085 | 0.224 | 104.596 | 0.380 | 0.705 |
| Ice-off_vs_Ice-on | Sporadic | 0.207 | 0.297 | 113.087 | 0.696 | 0.488 |
| Summer_vs_Ice-on | Sporadic | 0.122 | 0.297 | 101.428 | 0.410 | 0.683 |
| Ice-off_vs_Summer | Discontinuous | -0.238 | 0.303 | 115.255 | -0.784 | 0.435 |
| Ice-off_vs_Ice-on | Discontinuous | -0.147 | 0.296 | 120.697 | -0.498 | 0.619 |
| Summer_vs_Ice-on | Discontinuous | 0.090 | 0.253 | 100.121 | 0.356 | 0.722 |
| Ice-off_vs_Summer | Continuous | -0.375 | 0.211 | 88.439 | -1.780 | 0.078 |
| Ice-off_vs_Ice-on | Continuous | -0.573 | 0.206 | 87.020 | -2.777 | 0.007* |
| Summer_vs_Ice-on | Continuous | -0.198 | 0.206 | 87.020 | -0.959 | 0.340 |

**Supplementary Table 4.** The annual C emission (CO_2_ + diffusive CH_4_) per unit of aquatic area (± interquartile range, IQR) and climate parameters across permafrost zones.

|  | **Permafrost zone** | | | |
| --- | --- | --- | --- | --- |
|  | **Isolated** | **Sporadic** | **Discontinuous** | **Continuous** |
| ***n*** | 20 | 19 | 18 | 16 |
| Annual C emission, kg C m^-2^ yr^-1^ | 0.13 ± 0.14 | 0.09 ± 0.08 | 0.32 ± 0.35 | 0.31 ± 0.18 |
| MAAT, °C | -1.17 | -2.10 | -3.84 | -4.87 |
| Ice-free period, days | 141 | 137 | 118 | 111 |
| Mean annual precipitation, mm yr^-1^ | 499.7 | 439.6 | 426.3 | 383.9 |

**Supplementary Table 5.** Statistical results of regression analyses. All variables were log_10_-transformed. For details on statistics see Statistical analysis.

|  | **Dependent variable** | | | | | | | | | | | |
| --- | --- | --- | --- | --- | --- | --- | --- | --- | --- | --- | --- | --- |
|  | **Annual C emission (CO_2_ + diffusive CH_4_), kg C m^-2^ yr^-1^** | | | | | | | | | | | |
| **Predictor variable** | **(1)** | **(2)** | **(3)** | **(4)** | **(5)** | **(6)** | **(7)** | **(8)** | **(9)** | **(10)** | **(11)** | **(12)** |
| Landsat area, m^2^ | 0.00 (0.01) |  |  |  |  |  |  |  |  |  |  |  |
| Average lake depth, m |  | 0.06** (0.03) |  |  |  |  |  |  |  |  |  |  |
| SUVA_254_ |  |  | 0.06 (0.13) |  |  |  |  |  |  |  |  |  |
| Surface water O_2_, mg L^-1^ |  |  |  | -0.89*** (0.14) |  |  |  |  |  |  |  |  |
| DOC, mg L^-1^ |  |  |  |  | 0.06 (0.05) |  |  |  |  |  |  |  |
| DIC, mg L^-1^ |  |  |  |  |  | 0.08*** (0.03) |  |  |  |  |  |  |
| pH |  |  |  |  |  |  | 0.27** (0.10) |  |  |  |  |  |
| PO_4_, μg P L^-1^ |  |  |  |  |  |  |  | 0.07* (0.04) |  |  |  |  |
| NO_3_, μg N L^-1^ |  |  |  |  |  |  |  |  | -0.00 (0.02) |  |  |  |
| NH_4_, μg N L^-1^ |  |  |  |  |  |  |  |  |  | -0.00 (0.01) |  |  |
| DIN, μg L^-1^ |  |  |  |  |  |  |  |  |  |  | -0.00 (0.01) |  |
| TP, μg L^-1^ |  |  |  |  |  |  |  |  |  |  |  | 0.07*** (0.02) |
| Intercept | 0.07** (0.04) | 0.09*** (0.01) | 0.05 (0.07) | 0.99*** (0.15) | 0.01  (0.05) | 0.10*** (0.01) | -0.11 (0.07) | 0.04** (0.02) | 0.08*** (0.01) | 0.08*** (0.02) | 0.08*** (0.02) | -0.02 (0.03) |
| Observations | 72 | 73 | 73 | 73 | 73 | 73 | 73 | 72 | 72 | 72 | 72 | 73 |
| R^2^ | 0.00 | 0.07 | 0.00 | 0.35 | 0.02 | 0.11 | 0.09 | 0.04 | 0.00 | 0.00 | 0.00 | 0.11 |
| Adjusted R^2^ | -0.01 | 0.06 | -0.01 | 0.34 | 0.01 | 0.09 | 0.08 | 0.02 | -0.01 | -0.01 | -0.01 | 0.09 |
| Residual Std. Error | 0.07 (df=70) | 0.07 (df=71) | 0.07 (df=71) | 0.06 (df=71) | 0.07 (df=71) | 0.07 (df=71) | 0.07 (df=71) | 0.07 (df=70) | 0.07 (df=70) | 0.07 (df=70) | 0.07 (df=70) | 0.07 (df=71) |
| F Statistics | 0.03  (df=1,70) | 5.33** (df=1,71) | 0.19 (df=1,71) | 38.35*** (df=1,71) | 1.44 (df=1,71) | 8.54*** (df=1,71) | 6.84** (df=1,71) | 2.82* (df=1,70) | 0.00 (df=1,70) | 0.03 (df=1,70) | 0.02 (df=1,70) | 8.42*** (df=1,71) |
| Note: |  |  |  |  |  |  |  |  |  | **p*<0.1; | ***p*<0.05; | ****p*<0.01 |

**Supplementary Table 6.** O_2_ concentrations at the sediment interface (± interquartile range, IQR) across permafrost zones. Note that sediment surface O_2_ concentrations are expressed as averages of seasons. Dash stands for “not applicable”. For details on sampling see Methods.

|  | **Permafrost zone** | | | | | | | | | | | |
| --- | --- | --- | --- | --- | --- | --- | --- | --- | --- | --- | --- | --- |
|  | **Isolated** | | | **Sporadic** | | | **Discontinuous** | | | **Continuous** | | |
|  | **100 cm depth** | **150 cm depth** | **200 cm depth** | **100 cm depth** | **150 cm depth** | **200 cm depth** | **100 cm depth** | **150 cm depth** | **200 cm depth** | **100 cm depth** | **150 cm depth** | **200 cm depth** |
| ***n*** | 35 | 12 | 4 | 7 | - | - | 23 | - | - | 41 | 26 | 15 |
| Sediment surface O_2_, mg L^-1^ | 7.34 (4.68) | 7.33 (2.99) | 7.14 (5.15) | 10.49 (2.51) | - | - | 9.29 (2.34) | - | - | 9.93 (1.75) | 8.80 (2.99) | 7.64 (3.24) |

**Supplementary Table 7.** Total C emission from WSL permafrost lakes based on different lake area estimates. For details see Methods.

|  | **Type of data** | **Lake area, km^2^** | **Lake coverage, %** | **C emission, Tg C yr^-1^** | **Uncertainty, Tg C yr^-1^** |
| --- | --- | --- | --- | --- | --- |
| Verpoorter et al., 2014 | Global database | 84323 | 8.03 | 17.90 | 3.80 |
| Messager et al., 2016 | Global database | 41594 | 3.96 | 8.83 | 1.87 |
| Polishchuk et al., 2017 | Satellite inventory | 59700 | 5.69 | 12.67 | 2.69 |
| Polishchuk et al., 2018 | Satellite inventory | 64000 | 6.10 | 13.59 | 2.88 |

**Supplementary Table 8.** Statistical results of orthogonal contrasts. Between-seasons comparison of *p*CO_2_ concentrations (log_10_-transformed, n=180). The star indicates statistically significant difference at 0.05 level. For details on statistics see Statistical analysis.

| **Contrast** | **Permafrost zone** | **Estimate** | **SE** | **df** | **t.ratio** | **p.value** |
| --- | --- | --- | --- | --- | --- | --- |
| Ice-off_vs_Summer | Isolated | -0.083 | 0.079 | 117.542 | -1.059 | 0.292 |
| Ice-off_vs_Ice-on | Isolated | -0.018 | 0.198 | 147.622 | -0.093 | 0.926 |
| Summer_vs_Ice-on | Isolated | 0.065 | 0.198 | 147.111 | 0.329 | 0.743 |
| Ice-off_vs_Summer | Sporadic | 0.230 | 0.081 | 127.000 | 2.826 | 0.005* |
| Ice-off_vs_Ice-on | Sporadic | 0.068 | 0.120 | 141.109 | 0.568 | 0.571 |
| Summer_vs_Ice-on | Sporadic | -0.162 | 0.122 | 134.508 | -1.330 | 0.186 |
| Ice-off_vs_Summer | Discontinuous | -0.357 | 0.083 | 120.356 | -4.309 | 0.000* |
| Ice-off_vs_Ice-on | Discontinuous | -0.140 | 0.083 | 120.356 | -1.683 | 0.095 |
| Summer_vs_Ice-on | Discontinuous | 0.218 | 0.084 | 115.135 | 2.584 | 0.011* |
| Ice-off_vs_Summer | Continuous | -0.534 | 0.084 | 115.135 | -6.335 | 0.000* |
| Ice-off_vs_Ice-on | Continuous | -0.273 | 0.084 | 115.135 | -3.240 | 0.002* |
| Summer_vs_Ice-on | Continuous | 0.261 | 0.084 | 115.135 | 3.095 | 0.002* |

**Supplementary Table 9.** Statistical results of orthogonal contrasts. Between-seasons comparison of dissolved CH_4_ concentrations (log_10_-transformed, n=155). The star indicates statistically significant difference at 0.05 level. For details on statistics see Statistical analysis.

| **Contrast** | **Permafrost zone** | **Estimate** | **SE** | **df** | **t.ratio** | **p.value** |
| --- | --- | --- | --- | --- | --- | --- |
| Ice-off_vs_Summer | Isolated | 0.000 | 0.110 | 93.515 | -0.002 | 0.998 |
| Ice-off_vs_Ice-on | Isolated | 0.003 | 0.275 | 120.110 | 0.012 | 0.990 |
| Summer_vs_Ice-on | Isolated | 0.004 | 0.275 | 119.646 | 0.013 | 0.989 |
| Ice-off_vs_Summer | Sporadic | -0.043 | 0.116 | 103.874 | -0.376 | 0.708 |
| Ice-off_vs_Ice-on | Sporadic | -0.089 | 0.166 | 114.788 | -0.534 | 0.595 |
| Summer_vs_Ice-on | Sporadic | -0.045 | 0.170 | 107.374 | -0.266 | 0.790 |
| Ice-off_vs_Summer | Discontinuous | -0.281 | 0.160 | 126.403 | -1.754 | 0.082 |
| Ice-off_vs_Ice-on | Discontinuous | 0.038 | 0.171 | 123.000 | 0.221 | 0.825 |
| Summer_vs_Ice-on | Discontinuous | 0.319 | 0.144 | 110.296 | 2.214 | 0.029* |
| Ice-off_vs_Summer | Continuous | -0.003 | 0.138 | 100.242 | -0.025 | 0.980 |
| Ice-off_vs_Ice-on | Continuous | -0.311 | 0.138 | 100.242 | -2.251 | 0.027* |
| Summer_vs_Ice-on | Continuous | -0.307 | 0.117 | 91.499 | -2.625 | 0.010* |

**Supplementary Table 10.** Statistical results of orthogonal contrasts. Between-seasons comparison of total C fluxes (cube root-transformed, n=158). The star indicates statistically significant difference at 0.05 level. For details on statistics see Statistical analysis.

| **Contrast** | **Permafrost zone** | **Estimate** | **SE** | **df** | **t.ratio** | **p.value** |
| --- | --- | --- | --- | --- | --- | --- |
| Ice-off_vs_Summer | Isolated | -0.185 | 0.112 | 106.075 | -1.656 | 0.101 |
| Ice-off_vs_Ice-on | Isolated | -0.398 | 0.255 | 124.755 | -1.563 | 0.121 |
| Summer_vs_Ice-on | Isolated | -0.213 | 0.255 | 124.135 | -0.836 | 0.405 |
| Ice-off_vs_Summer | Sporadic | 0.193 | 0.126 | 124.776 | 1.530 | 0.129 |
| Ice-off_vs_Ice-on | Sporadic | -0.126 | 0.165 | 131.139 | -0.766 | 0.445 |
| Summer_vs_Ice-on | Sporadic | -0.319 | 0.163 | 121.691 | -1.959 | 0.052 |
| Ice-off_vs_Summer | Discontinuous | -0.477 | 0.108 | 103.775 | -4.411 | 0.000* |
| Ice-off_vs_Ice-on | Discontinuous | -0.388 | 0.108 | 103.775 | -3.587 | 0.001* |
| Summer_vs_Ice-on | Discontinuous | 0.089 | 0.112 | 100.801 | 0.795 | 0.428 |
| Ice-off_vs_Summer | Continuous | -0.683 | 0.119 | 103.506 | -5.756 | 0.000* |
| Ice-off_vs_Ice-on | Continuous | -0.293 | 0.107 | 98.194 | -2.723 | 0.008* |
| Summer_vs_Ice-on | Continuous | 0.391 | 0.119 | 103.506 | 3.291 | 0.001* |

**Supplementary References.**

1. Cole, J. J. & Caraco, N. F. Atmospheric exchange of carbon dioxide in a low-wind oligotrophic lake measured by the addition of SF 6. *Limnol. Oceanogr.* **43,** 647–656 (1998).

2. Vachon, D. & Prairie, Y. T. The ecosystem size and shape dependence of gas transfer velocity versus wind speed relationships in lakes. *Can. J. Fish. Aquat. Sci.* **70,** 1757–1764 (2013).
