# Supplementary material for: High carbon emissions from thermokarst lakes of Western Siberia
Source: Nat Commun. 2019 Apr 4;10:1552. doi: 10.1038/s41467-019-09592-1 (PMC6449335; doi:10.1038/s41467-019-09592-1)
Supplement: Supplementary file 2 — Description of Additional Supplementary Files [file 41467_2019_9592_MOESM2_ESM.pdf]

Description of Additional Supplementary Information

File Name: Supplementary Dataset 1
Description: Dataset created and analyzed in this study. “NA” stands for missing values whereas “SD” represents standard deviation of the mean.

File Name: Supplementary Dataset 2
Description: List of all chamber measurements and flux estimates for each of the sampled lakes across seasons. “NA” stands for missing values
